# Supplementary material for: Microbiological Epidemiology of Invasive Infections Due to Non-Beta-Hemolytic Streptococci, France, 2021
Source: Microbiol Spectr. 2023 May 18;11(3):e00160-23. doi: 10.1128/spectrum.00160-23 (PMC10269528; doi:10.1128/spectrum.00160-23)
Supplement: Supplemental file 1 — Figure S1. Download spectrum.00160-23-s0001.pdf, PDF file, 3.8 MB [file spectrum.00160-23-s0001.pdf]

A Benzylpenicillin BMD MIC (rows: Benzylpenicillin gradient test MIC)

Benzylpenicillin gradient test MIC	≤0,008	0,016	0,03	0,06	0,125	0,25	0,5	1	2	4	>4
≤0,008	3	6		3							
0,016	10	9	2	6							
0,03	10	24	21	29	6						
0,06	5	6	21	90	23	1					
0,125			3	36	56	14					
0,25					6	31	9				
0,5							16	9			
1							2	12	3		
2								4	7		
4									2	4	
>4										7	9

B Amoxicillin BMD MIC (rows: Amoxicillin gradient test MIC)

Amoxicillin gradient test MIC	≤0,008	0,016	0,03	0,06	0,125	0,25	0,5	1	2	4	>4
≤0,008	1	2	2	1		1					
0,016		7	3		1						
0,03	1	2	25	20	6						
0,06		2	11	58	17	1					
0,125	2	3	3	22	83	16	2				
0,25	1		1	7	29	36	10				
0,5				1	1	24	14	15			
1							7	11	7		
2								7	16	2	
4									1	4	1
>4										6	12

C Cefotaxime BMD MIC (rows: Cefotaxime gradient test MIC)

Cefotaxime gradient test MIC	≤0,008	0,016	0,03	0,06	0,125	0,25	0,5	1	2	4	>4
≤0,008	2	6	2								
0,016		4	10	3							
0,03	1	1	14	19	5	1					
0,06		2	8	49	30	6					
0,125		2	1	18	86	38	6				
0,25			1	2	15	93	24				
0,5						5	12	10	1		
1								3	4	2	
2									4	3	1
4											
>4									1	4	6

D Ceftriaxone BMD MIC (rows: Ceftriaxone gradient test MIC)

Ceftriaxone gradient test MIC	≤0,008	0,016	0,03	0,06	0,125	0,25	0,5	1	2	4	>4
≤0,008		2	2	1							
0,016		3	9	8	3	1					
0,03			8	27	6	2	1				
0,06			3	15	51	11	3				
0,125		2	1	8	39	79	22				
0,25			1	1	7	37	87	11			
0,5					1	2	6	10	4	1	
1								1	10	4	
2									1	2	1
4										2	1
>4										2	6

Figure S1. Correlation between MIC values determined by broth microdilution and gradient Etests for 505 non-beta-haemolytic Streptococcus invasive strains.

MIC values of benzylpenicillin (A), amoxicillin (B), cefotaxime (C) and ceftriaxone (D) were determined for all the isolates by broth microdilution (BMD) using a custom microtiter plate (SWE1SPEC, ThermoFisher) and by gradient Etest and interpreted according to EUCAST recommendations (v 13.0).
