# Supplementary material for: Microbiological Epidemiology of Invasive Infections Due to Non-Beta-Hemolytic Streptococci, France, 2021
Source: Microbiol Spectr. 2023 May 18;11(3):e00160-23. doi: 10.1128/spectrum.00160-23 (PMC10269528; doi:10.1128/spectrum.00160-23)
Supplement: Supplemental file 2 — Figure S2. Download spectrum.00160-23-s0002.pdf, PDF file, 3.8 MB [file spectrum.00160-23-s0002.pdf]

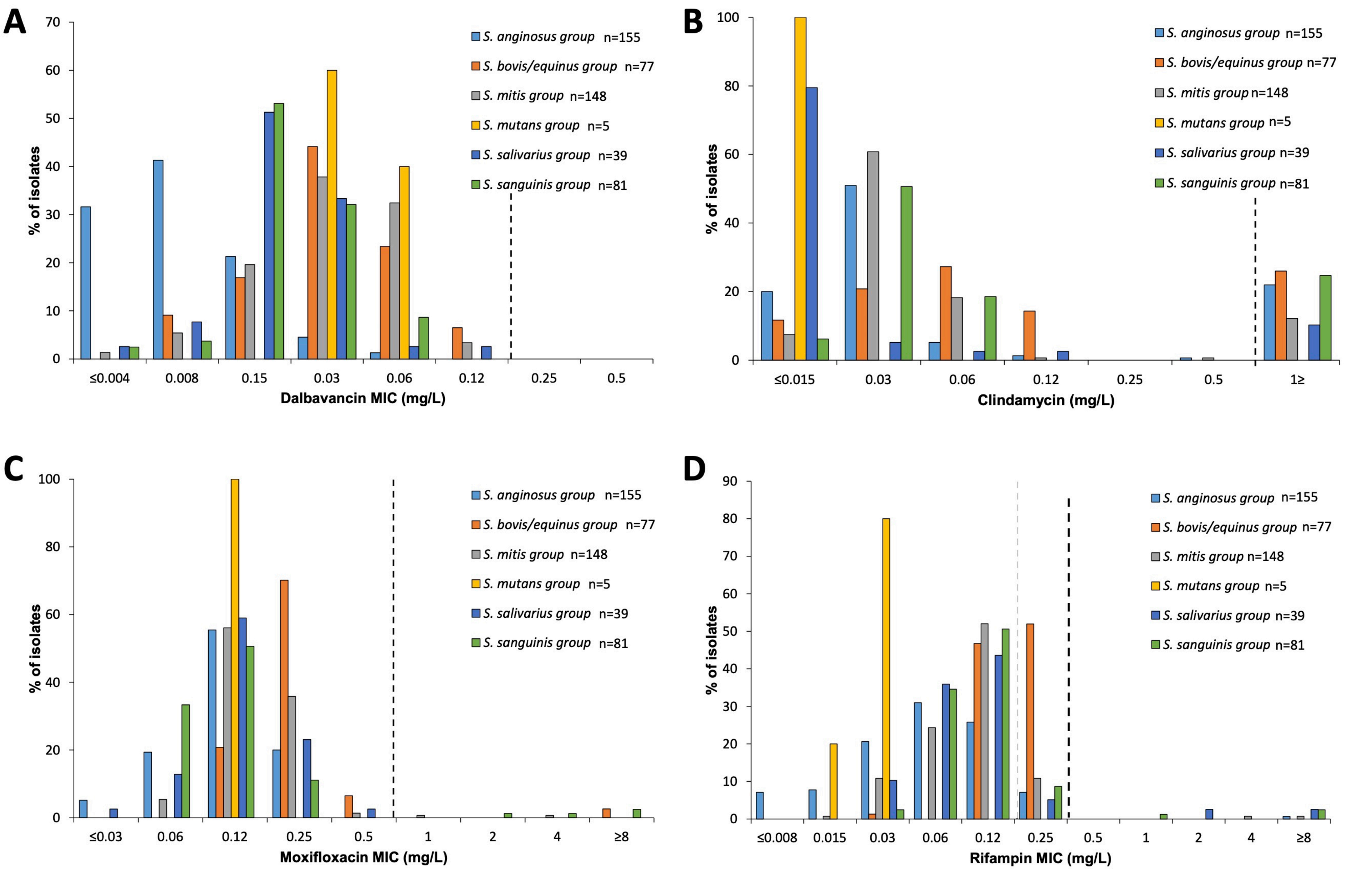

Figure S2. Distribution of dalbavancin (A), clindamycin (B), moxifloxacin (C) and rifampin (D) MIC values within each non-beta-haemolytic streptococcal group (n=505).

MIC values were determined by broth microdilution. The dotted lines indicate the EUCAST v 13.0 clinical breakpoints (A, B), and the ECOFFs (C, D), dalbavancin clinical breakpoint being only applicable to isolates of the *anginosus* group.
