# Supplementary material for: Microbiological Epidemiology of Invasive Infections Due to Non-Beta-Hemolytic Streptococci, France, 2021
Source: Microbiol Spectr. 2023 May 18;11(3):e00160-23. doi: 10.1128/spectrum.00160-23 (PMC10269528; doi:10.1128/spectrum.00160-23)
Supplement: Supplemental file 3 — Tables S1-S10. Download spectrum.00160-23-s0003.pdf, PDF file, 0.2 MB [file spectrum.00160-23-s0003.pdf]

Table S1. Clinical manifestations of the 522 NHBS invasive infections.

Streptococcus group	Bacteraemia without focus	Intra-abdominal infections	Endocarditis	Pulmonary infections	Bone and joint infections	Skin and soft tissue infections	Urinary tract infections	Upper respiratory tract infections	Central nervous system infections	Others[a]	Total, n (%)
anginosus	**58**	**43**	**3**	**26**	**13**	**14**	**5**	**6**	**2**	**2**	**172 (33.0%)**
S. anginosus	33	30	3	10	6	8	4	1	0	2	97 (18.6%)
S. constellatus	22	12	0	7	5	3	1	2	1	0	53 (10.2%)
S. intermedius	3	1	0	9	2	3		3	1	0	22 (4.2%)
bovis/equinus	**43**	**11**	**12**	**1**	**3**	**2**	**2**	**0**	**2**	**1**	**77 (14.8%)**
S. gallolyticus	16	3	8	0	1	0	1	0	1	0	30 (5.7%)
S. infantarius	7	1	1	0	0	1	0	0	0	0	10 (1.9%)
S. lutetiensis	7	4	1	0	1	0	0	0	0	0	13 (2.5%)
S. pasteurianus	13	3	2	1	1	1	1	0	1	1	24 (4.6%)
mitis	**78**	**16**	**20**	**15**	**7**	**3**	**3**	**4**	**0**	**2**	**148 (28.4%)**
S. australis	2	2	0	0	0	0	0	0	0	0	4 (0.8%)
S. cristatus	2	3	1	0	1	0	0	0	0	0	7 (1.3%)
S. infantis	1	1	0	0	0	0	0	1	0	0	3 (0.6%)
S. massiliensis	1	0	0	1	0	0	0	0	0	1	3 (0.6%)
S. mitis	26	2	4	3	0	1	1	1	0	0	38 (7.3%)
S. oralis	45	6	14	6	6	1	2	2	0	1	83 (15.9%)
S. peroris	1	1	0	2	0	0	0	0	0	0	4 (0.8%)
S. pseudopneumoniae	0	0	1	3	0	1	0	0	0	0	5 (1.0%)
S. sinensis	0	1	0	0	0	0	0	0	0	0	1 (0.2%)
mutans	**3**	**0**	**2**	**0**	**0**	**0**	**0**	**0**	**0**	**0**	**5 (1.0%)**
S. mutans	3	0	2	0	0	0	0	0	0	0	5 (1.0%)
salivarius	**21**	**10**	**2**	**3**	**0**	**1**	**0**	**0**	**2**	**0**	**39 (7.5%)**
S. salivarius	16	7	2	2	0	1	0	0	2	0	30 (5.7%)
S. thermophilus	1	0	0	0	0	0	0	0	0	0	1 (0.2%)
S. vestibularis	4	3	0	1	0	0	0	0	0	0	8 (1.5%)
sanguinis	**38**	**15**	**19**	**6**	**3**	**0**	**0**	**0**	**0**	**0**	**81 (15.5%)**
S. gordonii	10	1	3	0	2	0	0	0	0	0	16 (3.1%)
S. parasanguinis	11	12	2	4	0	0	0	0	0	0	29 (5.6%)
S. sanguinis	17	2	14	2	1	0	0	0	0	0	36 (6.9%)
**Total, n (%)**	**241 (46.2%)**	**95 (18.2%)**	**58 (11.1%)**	**51 (9.8%)**	**26 (5.0%)**	**20 (3.8%)**	**10 (1.9%)**	**10 (1.9%)**	**6 (1.1%)**	**5 (1.0%)**	**522 (100%)**

[a] including vascular and catheter-related bloodstream infections (n = 3), endophthalmitis (n = 1) and upper genital infection (n = 1).

Table S2. Comparison of MIC values obtained by BMD and gradient Etest on 505 NBHS.

Agreement	Benzylpenicillin	Amoxicillin	Cefotaxime	Ceftriaxone
**Categorical agreement**	97.8%	95.0%	97.8%	94.9%
Minor errors	2.2%	5.0%	NA	NA
Major errors	0%	0%	NA	NA
Very major errors	0%	0%	2.2%	5.1%
**Essential agreement**	95.1%	94.1%	92.9%	82.9%
**Absolute agreement**	54.4%	53.5%	55.0%	22.9%
**Bias**	1.1%	2.9%	24.3%	68.6%

NA: not applicable.

Interpretation according to EUCAST guidelines v 13.0.

Table S3. Beta-lactam resistance phenotypes of the 522 NBHS.

	***Streptococcus* group**					
**Beta-lactam resistance phenotype, n (%)**	***anginosus* n=172**	***bovis/equinus* n=77**	***mitis* n=148**	***mutans* n=5**	***salivarius* n=39**	***sanguinis* n=81**
Susceptible	172 (100%)	77 (100%)	102 (68.9%)	5 (100%)	28 (71.8%)	39 (48.1%)
Benzylpenicillin	0	0	1 (0.7)	0	2 (5.1%)	0
Benzylpenicillin + aminopenicillins	0	0	8 (5.4%)	0	4 (10.3%)	17 (43.6%)
Benzylpenicillin + aminopenicillins + 3rd generation cephalosporins	0	0	32 (21.6%)	0	4 (10.3%)	15 (18.5%)
Benzylpenicillin + 3rd generation cephalosporins	0	0	1 (0.7%)	0	0	0
Aminopenicillins	0	0	0	0	0	9 (11.1%)
3rd generation cephalosporin	0	0	4 (2.7%)	0	1 (2.6%)	1 (1.2%)

Values indicated the number (%) of isolates displaying intermediate susceptibility or resistance to benzylpenicillin and aminopenicillins (amoxicillin or ampicillin), and resistance to 3rd generation cephalosporins (cefotaxime or ceftriaxone) according to EUCAST v 13.0 breakpoints.

Table S4. Performances of the 1-unit PEN-disk screening for the detection of decreased-susceptibility to beta-lactams.

***Streptococcus* group (n)**	**Sensitivity**		**Specificity**		**Positive predictive value**		**Negative predictive value**	
**Diameter breakpoint[a]**	**18 mm**	**21 mm**	**18 mm**	**21 mm**	**18 mm**	**21 mm**	**18 mm**	**21 mm**
mitis (148)	69.6 %	87.0 %	100 %	92.2 %	100 %	83.3 %	87.9 %	94.0 %
salivarius (39)	72.7 %	81.8 %	100 %	75.0 %	100 %	56.3 %	90.3 %	91.3 %
sanguinis (81)	45.2 %	69.0 %	100 %	97.4 %	100 %	96.7 %	62.9 %	74.5 %
**Total isolates (n=522)**	**59.6 %**	**78.8 %**	**100 %**	**95.0 %**	**100 %**	**78.8 %**	**91.0 %**	**95.0 %**

[a] Susceptible breakpoints from EUCAST v 12.0 (18 mm) and EUCAST v 13.0 (21 mm).

PEN, benzylpenicillin.

Table S5. Beta-lactam resistance phenotypes of the 40 NBHS isolates falsely categorised as susceptible by the 1-unit PEN disk screening (susceptible breakpoint 18 mm, EUCAST v12.0).

**Phenotype [a]**				***Streptococcus* group**		
**Benzylpenicillin**	**Aminopenicillins (ampicillin or amoxicillin)**	**Third-generation cephalosporins (cefotaxime or ceftriaxone)**	**Isolates n (%)**	***mitis*** 14 (35%)	***salivarius*** 3 (7%)	***sanguinis*** 23 (58%)
I	S	S	2 (5 %)	0	2	0
I	I	S	14 (35 %)	3	0	11
I	I	R	8 (20 %)	6	0	2
I	S	R	1 (2 %)	1	0	0
S	S	R	6 (15 %)	4	1	1
S	I	S	9 (23 %)	0	0	9

[a] MIC values determined by broth microdilution.

NBHS, non-beta-haemolytic streptococci; S, susceptible; I, susceptible increased exposure; R, resistant.

Table S6. Beta-lactam resistance phenotypes of the 21 NBHS isolates falsely categorised as susceptible by the 1-unit PEN disk screening (susceptible breakpoint 21 mm, EUCAST v13.0).

Phenotype [a]			Isolates	*Streptococcus* group		
**Benzylpenicillin**	**Aminopenicillins (ampicillin or amoxicillin)**	**Third-generation cephalosporins (cefotaxime or ceftriaxone)**	**n (%)**	***mitis*** 6 (28.6%)	***salivarius*** 2 (9.5%)	***sanguinis*** 13 (61.9%)
I	S	S	2 (9.5%)	0	2	0
I	I	S	4 (19.0%)	1	0	3
I	I	R	2 (9.5%)	1	0	1
I	S	R	1 (4.8%)	1	0	0
S	S	R	4 (19.0%)	3	0	1
S	I	S	8 (38.1%)	0	0	8

[a] MIC values determined by broth microdilution.

NBHS, non-beta-haemolytic streptococci; S, susceptible; I, susceptible increased exposure; R, resistant.

Table S7. Clinical characteristics of the 79 NBHS invasive infections in 2022.

Streptococcus group	*anginosus*	*bovis/equinus*	*mitis*	*salivarius*	*sanguinis*	Total
**Sex ratio M/F**	1.6	0.9	1.6	(4 males)	2.5	1.5
**Age, median**	50 y	77 y	57 y	69 y	85 y	65 y
(min – max)	(0 d - 97 y)	(0 d - 100 y)	(11 - 95 y)	(65 - 87 y)	(11 - 98 y)	(0 d - 100 y)
**Age, interquartile range, in years**	31 - 69	62 - 86	40 - 70	ND	49 - 93	43 - 81
**Total, n (%)**	**26 (33%)**	**21 (27%)**	**21 (27%)**	**4 (5%)**	**7 (8%)**	**79 (100%)**
**Adult cases (≥ 18 y), n (%)[a]**	**24 (92%)**	**19 (90%)**	**20 (95%)**	**4**	**6**	**73 (92%)**
Bacteremia without focus	2 (8%)	12 (57%)	5 (24%)	3	1	23 (29%)
Intra-abdominal infections	5 (19%)	0	5 (24%)	0	0	10 (13%)
Endocarditis	1 (4%)	4 (19%)	2 (10%)	0	1	8 (10%)
Pulmonary infections	4 (15%)	0	2 (10%)	0	2	8 (10%°
Bone and joint infections	2 (8%)	3 (14%)	2 (10%)	1	1	9 (11%)
Skin and soft tissue infections	1 (4%)	0	2 (10%)	0	0	3 (4%)
Upper respiratory tract infections	4 (15%)	0	2 (10%)	0	0	6 (8%)
Central nervous system infections	1 (4%)	0	0	0	0	1 (1%)
Others[b]	4 (15%)	0	0	0	1	5 (6%)
**Neonates and infants (birth - ≤ 1 y), n (%)[a]**	**1 (4%)**	**2 (10%)**	**0**	**0**	**0**	**3 (4%)**
Early Onset Sepsis (0-3 d)	1 (4%)	2 (10%)	0	0	0	3 (4%)
**Pediatric cases (1 - 18 y), n (%)[a]**	**1 (4%)**	**0**	**1 (5%)**	**0**	**1**	**3 (4%)**
Bacteremia without focus	0	0	1 (5%)	0	0	1 (1%)
Others[c]	1 (4%)	0	0	0	1	2 (3%)

[a] Percentages are relative to the total isolates within each *Streptococcus* group and were not calculated for the *S. salivarius* and *S. sanguinis* groups because of the small number of cases.

[b] Including upper -genital infections (n = 4) and urinary tract infections (n = 1).

[c] Including skin and soft tissue infection (n = 1), upper respiratory tract infections (n = 1).

d, days; m, months; y, years; ND, not determined.

Table S8. Beta-lactam agents - Susceptibility and MIC distributions of 79 invasive NBHS isolates in 2022.

		MIC[a]									MIC interpretation[b], n (%)		
***Streptococcus* group (n; %)**	**Antimicrobial agent**	**≤0.016**	**0.032**	**0.064**	**0.125**	**0.25**	**0.5**	**1**	**2**	**≥4**	**S**	**I**	**R**
***anginosus* (26; 33%)**	Benzylpenicillin	0	7	10	7	2	0	0	0	0	26 (100%)	0	0
	Amoxicillin	2	2	3	7	10	2	0	0	0	26 (100%)	0	0
	Cefotaxime	1	0	4	4	16	1	0	0	0	26 (100%)	NA	0
	Ceftriaxone	0	0	3	8	14	1	0	0	0	26 (100%)	NA	0
***bovis/equinus* (21; 27%)**	Benzylpenicillin	0	0	8	9	4	0	0	0	0	21 (100%)	0	0
	Amoxicillin	0	1	2	11	7	0	0	0	0	21 (100%)	0	0
	Cefotaxime	0	0	3	8	10	0	0	0	0	21 (100%)	NA	0
	Ceftriaxone	1	2	0	15	3	0	0	0	0	21 (100%)	NA	0
***mitis* (21; 27%)**	Benzylpenicillin	0	1	1	3	2	0	4	0	10	7 (33%)	4 (19%)	10 (48%)
	Amoxicillin	0	1	1	2	1	3	2	1	10	8 (38%)	3 (14%)	10 (48%)
	Cefotaxime	0	0	1	2	3	2	1	1	11	8 (38%)	NA	13 (62%)
	Ceftriaxone	0	1	1	2	3	2	0	3	9	9 (43%)	NA	12 (57%)
***salivarius* (4; 5%)**	Benzylpenicillin	0	0	1	1	1	1	0	0	0	3 (75%)	1 (25%)	0
	Amoxicillin	0	0	1	1	0	0	2	0	0	2 (50%)	2 (50%)	0
	Cefotaxime	1	0	1	0	1	0	1	0	0	3 (75%)	NA	1 (25%)
	Ceftriaxone	0	1	1	0	1	1	0	0	0	4 (100%)	NA	0
***sanguinis* (7; 8%)**	Benzylpenicillin	2	0	2	1	0	0	1	0	1	5 (71%)	1 (14.5%)	1 (14.5%)
	Amoxicillin	0	1	0	2	2	0	0	1	1	5 (71%)	1 (14.5%)	1 (14.5%)
	Cefotaxime	1	1	3	0	0	1	0	0	1	6 (86%)	NA	1 (14%)
	Ceftriaxone	0	2	3	0	1	0	0	0	1	6 (86%)	NA	1 (14%)

[a] MICs determined by gradient Etest.

[b] MICs interpretation according to EUCAST v 13.0. Susceptible isolates in shaded area, resistant isolates underlined.

NA, not applicable; S, susceptible; I, susceptible increased exposure; R, resistant.

Table S9. Macrolides, lincosamides and streptogramins (MLS) – Susceptibility, resistance phenotypes and genotypes of the 79 invasive NBHS isolates in 2022.

***Streptococcus* group**	**Resistant isolates, n (%)**	**Disk diffusion phenotype[a,b], n (%)**				**Resistance determinants, n (%)[b]**					
		**L/LS$_A$**	**M**	**iMLS$_B$**	**cMLS$_B$**	***mef***	***erm*(A)**	***erm*(B)**	***erm*(T)**	***lsa*(A)**	***lsa*(B)**
anginosus (n=26)	12 (46%)	2 (17%)	0 (0%)	2 (17%)	8 (67%)	0 (0%)	4 (33%)	5 (42%)	1 (8%)	3 (25%)	2 (17%)
bovis/equinus (n=21)	13 (62%)	3 (23%)	0 (0%)	0 (0%)	10 (77%)	0 (0%)	2 (15%)	7 (54%)	1 (8%)	0 (0%)	1 (8%)
mitis (n=21)	13 (62%)	0 (0%)	10 (77%)	0 (0%)	3 (23%)	12 (92%)	1 (8%)	2 (15%)	0 (0%)	0 (0%)	0 (0%)
salivarius (n=4)	2 (50%)	0 (0%)	1 (50%)	0 (0%)	1 (50%)	1 (50%)	1 (50%)	0 (0%)	0 (0%)	0 (0%)	0 (0%)
sanguinis (n=7)	5 (71%)	1 (20%)	3 (60%)	0 (0%)	1 (20%)	3 (60%)	1 (20%)	0 (0%)	0 (0%)	1 (20%)	1 (20%)
Total (n=79)	45 (57%)	6 (13%)	14 (31%)	2 (4%)	23 (51%)	16 (36%)	9 (20%)	14 (31%)	2 (4%)	4 (9%)	4 (9%)

[a] DD phenotypes were interpreted according to the guidelines on Antibiotic Susceptibility Testing of the French Society for Microbiology (CA-SFM).

[b] Percentages are relative to total resistant isolates within each group.

L, resistance to lincosamides; LS$_A$, resistance to lincosamides and streptogramin A; M, efflux-mediated resistance to C14 and C15 macrolides; iMLSB, inducible resistance to macrolides, lincosamides and streptogramin B; cMLS$_B$, constitutive resistance to macrolides, lincosamides and streptogramin B; NA: not applicable.

Table S10. Tetracyclines - Susceptibility and resistance determinants of the 79 invasive NBHS isolates in 2022[a].

***Streptococcus* group**	**Resistant isolates, n (%)**	**Resistance determinant, n (%)[b]**		
		***tet*(M)**	***tet*(L)**	***tet*(O)**
anginosus, n=26	10 (38%)	7 (70%)	1 (10%)	4 (40%)
bovis/equinus, n=21	17 (81%)	13 (76%)	8 (47%)	4 (24%)
mitis, n=21	5 (24%)	5 (100%)	0 (0%)	0 (0%)
salivarius, n=4	0 (0%)	-	-	-
sanguinis, n=7	0 (0%)	-	-	-
Total, n=79	32 (41%)	25 (78%)	9 (28%)	8 (25%)

[a] Interpreted according to the guidelines on Antibiotic Susceptibility Testing of the French Society for Microbiology.

[b] Percentages are relative to total resistant isolates within each group.
